# Recent Advances in the Brain–Gut Axis in the Development and Progression of Depression

**DOI:** 10.62641/aep.v54i4.2252

**Published:** 2026-08-15

**Authors:** Meinan Chen, Yanlai Xu

**Affiliations:** ^1^Department of Traditional Chinese Medicine, Qingdao Special Service Sanatorium of PLA Navy, 266071 Qingdao, Shandong, China

**Keywords:** depressive disorder, gastrointestinal microbiome, brain–gut axis, hypothalamic–pituitary–adrenal axis

## Abstract

The prevalence of depression is increasing year by year. Accumulating evidence has shown that the brain–gut axis (BGA) may contribute largely to the development and progression of depression. Gut microbial community composition and metabolites may contribute to the pathophysiological mechanisms of depression by influencing bidirectional communication with the BGA. Gut microbiota dysbiosis has been implicated in the pathogenesis of depression and may provide new potential targets for therapeutic interventions. This article reviews current academic investigations into the association between BGA and depression and discusses how to improve depressive symptoms through the BGA, seeking to offer insights for future research and clinical treatment strategies for depression.

## Introduction

Depression is a common and persistent mental disorder in clinical practice. It can occur at any age, including children, adolescents, and older adults. Core symptoms of depression include low mood, anhedonia, decreased self-esteem, irritability, and sleep disturbances [1, 2]. According to the World Health Organization (WHO), over 300 million people worldwide are living with depression, and it is estimated that by 2030, depression will surpass chronic cardiovascular disease to rank as the leading contributor to the worldwide disease burden [3, 4]. Studies have shown that nearly 50% of patients with depression do not receive adequate treatment, and the proportion is even higher in some low- and middle-income countries [5].

The monoamine hypothesis remains one of the most widely accepted theories of depression. The hypothesis proposes that depression is associated with the depletion of serotonin, norepinephrine, and dopamine in the central nervous system. Moreover, this hypothesis underpins the theoretical framework for the development of antidepressant drugs (e.g., selective serotonin reuptake inhibitors [SSRI]) [6]. Nevertheless, nearly 30% of patients with major depressive disorder fail to achieve a clinical response to monoamine antidepressants [7]. Consequently, there may be other mechanisms that lead to depression, and exploring these mechanisms may provide potential targets for novel therapeutic strategies.

In recent studies, the association between gut microbiota and the central nervous system has provided new insights into neuropsychiatric disorders. The brain–gut axis (BGA) functions as a bidirectional communication network connecting the enteric nervous system and the central nervous system. Although the gut and brain are anatomically separate, they communicate through multiple pathways, such as the production of neuroactive substances, metabolites and hormones, the immune system, vagus nerve signalling, and hypothalamic–pituitary–adrenal (HPA) axis activation [8]. Increasing evidence suggests that antidepressant treatments may affect depression symptoms by indirectly regulating the composition of gut microbiota, improving intestinal barrier function and regulating microbiota metabolites, further suggesting the potential role of the BGA in treating depressive disorders [9].

Although previous reviews have mainly focused on the mechanisms underlying the BGA, but have not comprehensively addressed intervention strategies based on the BGA. Based on a comprehensive search of clinical studies, experimental studies, and recent reviews, this article widely includes diverse study types and up-to-date evidence. It not only summarizes the association mechanisms between the BGA and depression, but also integrates a variety of interventions including probiotics, fecal microbiota transplantation (FMT), medications, diet, and physical exercise. By integrating current evidence on both mechanisms and interventions, this review aims to provide a broader perspective on the clinical prevention and treatment of depression.

## Literature Search and Study Selection

To collect relevant research literature, we searched the PubMed/MEDLINE, Scopus and Web of Science databases, with no restrictions on publication language and publication type. Search terms included “depression”, “gut microbiota” and “BGA”. The retrieval time was up to April 2026. Microsoft Excel was used to manage and sort out the retrieved references, and duplicate records were removed prior to screening.

Study selection was performed independently by two reviewers to ensure the objectivity and accuracy of the screening results. First, duplicate documents were removed; then, the reviewers screened the retrieved articles by reading titles and abstracts, excluding articles that were not related to the research topic. Finally, the remaining articles were subjected to full-text screening to determine their eligibility for inclusion, and the final included studies were determined.

Studies were eligible if they focused on the relationship between the BGA and the development and progression of depression, including original studies, systematic reviews, and meta-analyses. Studies that did not address the core research topic or were case reports were excluded.

For each included study, key information was extracted independently by the two reviewers, including author information, publication date, study type (human or animal studies), research methods, and main research results related to the BGA and depression.

## Characteristics of Included Studies

This review include studies related to gut microbiota, BGA, and depression. The included literature covered various types of research including reviews, systematic reviews, Mendelian randomization studies, randomized controlled trials, animal experiments, and meta-analyses. The included studies were published between 2017 and 2025, mainly concentrating on the recent 5 years (2020–2025), reflecting growing interest in this field. The characteristics and principal findings of the included studies are summarized in Table 1 (Ref. [8, 9, 10, 11, 12, 13, 14, 15, 16, 17]).

**Table 1.** **Summary of key findings from the included studies**.

Author/Year	Reference	Study design	Key findings
Góralczyk-Bińkowska *et al*. 2022	[8]	Review	Gut microbiota dysbiosis is involved in the pathogenesis of depression, schizophrenia and other psychiatric disorders via multiple pathways including metabolism, immunity and neural signaling, suggesting that targeting microbial homeostasis may provide potential adjunctive strategies for psychiatric disorders.
Socała *et al*. 2021	[9]	Review	This review elucidated the core regulatory role of the BGA in neuropsychiatric disorders and revealed the cascade effects among microbial metabolites, neuroinflammation and the HPA axis, providing a theoretical framework for gut-derived interventions.
Cao *et al*. 2025	[10]	Systematic review	Based on 24 case-control studies, inconsistent results were observed regarding gut microbiota diversity in patients with depression and anxiety, while the common characteristics were enrichment of pro-inflammatory bacteria and depletion of SCFA-producing anti-inflammatory bacteria.
Lai and Xiong 2025	[11]	Mendelian randomization study	Specific gut microbiota such as *Bifidobacterium* and *Actinomycetes* were associated with a reduced risk of depression, whereas elevated abundance of certain microbiota increased depression risk. No reverse causal effect of depression on gut microbiota composition was identified.
Schaub *et al*. 2022	[12]	Randomized controlled trial	A multicenter, double-blind RCT confirmed that short-term high-dose multi-strain probiotics significantly ameliorated depressive symptoms, preserved gut microbiota diversity, increased *Lactobacillus* abundance, reduced amygdala activation in response to neutral faces, and modulated depressive mood.
Nikolova *et al*. 2023	[13]	Randomized controlled trial	The BGA bidirectionally regulates mood through immune, metabolic and neural pathways, representing a key mechanism in depression pathogenesis. Probiotics can modulate gut microbiota and improve BGA function. As an adjunct to antidepressants, they significantly alleviate depressive and anxiety symptoms with favorable safety and adherence profiles.
Jiang *et al*. 2024	[14]	Randomized controlled trial	Fecal microbiota transplantation (FMT) significantly improved depressive symptoms by regulating gut microbiota through the BGA and affecting neuropsychiatric manifestations. It also relieved abnormal liver function indicators, which may contribute to alleviating related systemic and persistent somatic symptoms.
Chen *et al*. 2025	[15]	Animal experiment	Pharmacological intervention alleviated depression-like behaviors in rats with post-stroke depression, mainly by regulating the microbiota-brain–gut axis, inhibiting neuroinflammation and modulating central neurotransmitter levels.
Jacka *et al*. 2017	[16]	Randomized controlled trial	The SMILES trial demonstrated that a 12-week modified Mediterranean diet significantly improved remission rates in patients with major depressive disorder, providing high-level evidence for dietary interventions in depression.
Pearce *et al*. 2022	[17]	Meta-analysis	This meta-analysis included 15 prospective cohort studies involving 191,130 adults, and confirmed that physical activity can effectively improve depression.

Abbreviations: BGA, brain–gut axis; HPA, hypothalamic–pituitary–adrenal; RCT, randomized controlled trial; SCFA, short-chain fatty acid.

## Overview of the BGA

The BGA represents a multi-faceted reciprocal signal exchange system that connects the enteric nervous system and the central nervous system. The BGA contains three key barrier structures: the intestinal epithelial barrier, the blood-brain barrier, and the blood–cerebrospinal fluid barrier. These barriers maintain the homeostasis of the internal environment in different compartments of the BGA, while the permeability of the barriers is regulated by a variety of factors such as the gut microbiota composition and immune inflammation [18]. According to the BGA regulatory network, the brain can mediate the reorganization of intestinal function and gut microbiota composition through the autonomic nervous system, affecting intestinal transport, peristalsis, secretion, and mucosal permeability, leading to gut microbiota dysbiosis and related diseases. Such regulatory pathways can significantly affect stress, anxiety, cognition, and neuropsychiatric disorders such as depression, bipolar disorder, schizophrenia, and anxiety [19, 20]. Among the mediators involved in the BGA, 5-hydroxytryptamine (5-HT) acts as an important mediator, exerting regulatory effects on gastrointestinal physiology and central neural activity, and represents an important neurotransmitter associated with gastrointestinal impairment and affective disorders including depression and anxiety [21].

**Fig. 1.**
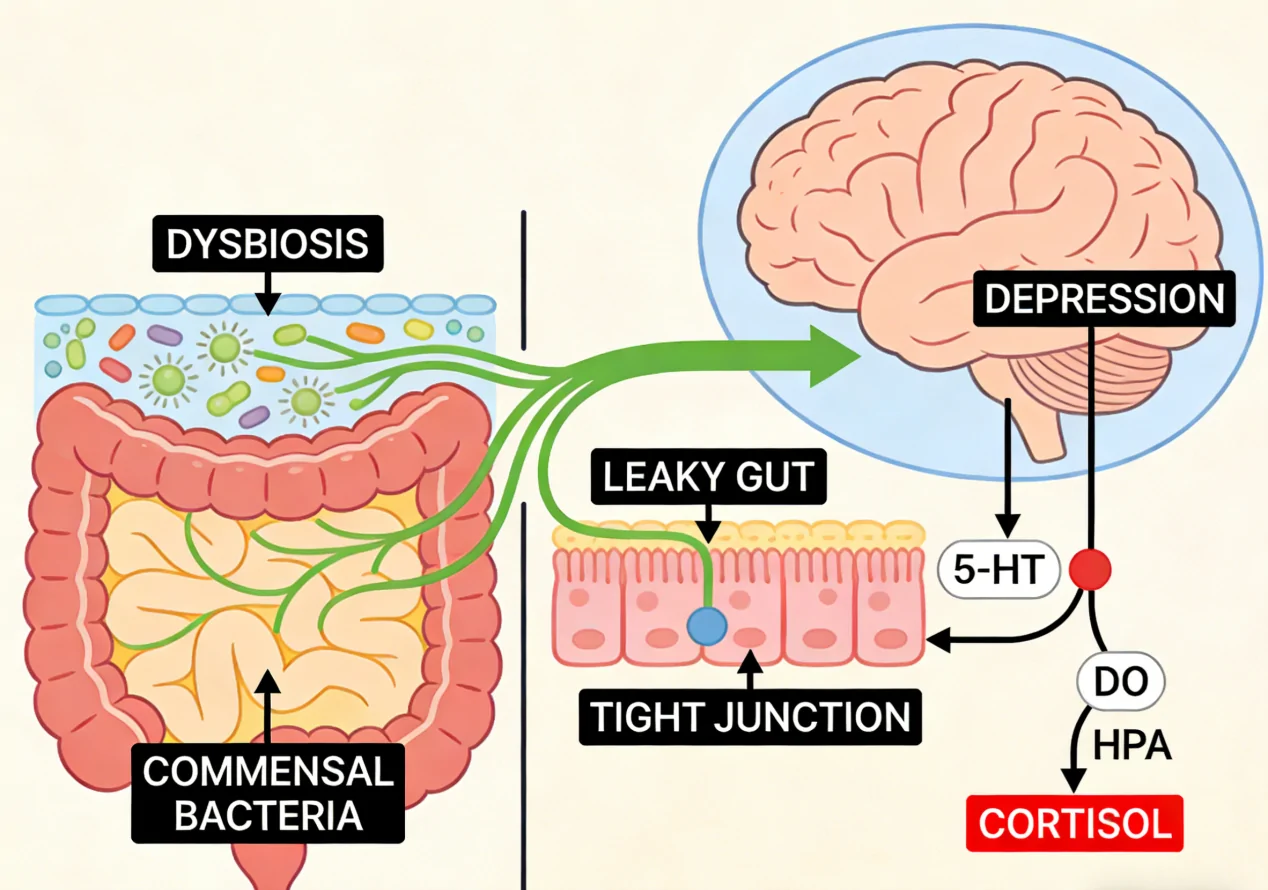
**The BGA establishes the connection between the enteric nervous system and the central nervous system**. Note: This figure presents a simplified overview to illustrate the key relationship between gut microbiota and central nervous system (CNS) regulation in depression. The term “gut microbiota dysbiosis” refers to the overall imbalance of intestinal microbial community, mainly manifested as increased pro-inflammatory bacteria and decreased anti-inflammatory and short-chain fatty acid (SCFA)-producing bacteria. The enteric nervous system (ENS) connects the gut with the CNS through neural, immune, metabolic and humoral pathways. For the sake of clarity and readability, the central effects shown on the right side are simplified to highlight major outcomes including disturbances of serotonin (5-HT) and dopamine (DO), neuroinflammation, and hyperactivity of the hypothalamic-pituitary-adrenal (HPA) axis. Detailed physiological pathways are comprehensively described in the main text. BGA, brain–gut axis.

Gut microbiota is a key component of the BGA. The human gut microbiota is a complex and highly diverse microbial community comprising bacteria, archaea, eukaryotes, viruses, parasites, and other eukaryotic microorganisms. An animal study has confirmed that the absence of gut microbiota during the growth period of mice may trigger dysregulated development of the enteric nervous system, thereby affecting intestinal peristalsis and undermines the integrity of the intestinal mucosal barrier [22]. The use of antibiotics in early childhood can easily cause gut microbiota dysbiosis, which not only leads to inflammatory bowel disease, but also increases the risk of neurodevelopmental abnormalities. This phenomenon further corroborates the regulatory link between gut microbiota and the central nervous system. Chidambaram *et al*. [23] reported that gut microbiota dysbiosis may impair the intestinal barrier and contribute to BGA dysregulation, potentially triggering autoimmunity, chronic neuroinflammation and ultimately promoting neurodegenerative development. The above studies have confirmed that the BGA is the core hub connecting the gastrointestinal tract and the nervous system. Further investigation of the relationship between the BGA and depression will open up new ideas and directions for clinical treatment. The connection between the enteric nervous system and the central nervous system mediated by the BGA is shown in Fig. 1.

## The Relationship Between BGA and Depression

### Characteristics of Gut Microbiota Dysbiosis in Depressive Disorders

Gut microbiota is a rich source of diverse metabolites. These metabolites contribute to bidirectional communication between the gut and central nervous system through the BGA [24]. Gut microbiota can generate monoamines, tryptophan metabolites, branched-chain amino acids and other metabolites [25]. These products may damage the intestinal epithelium and trigger local inflammation. The damage of the intestinal barrier function may increase intestinal permeability, which can further trigger systemic inflammatory response and affect brain function. This is an important inducing factor for the development of depression. In addition, gut microbiota also participates in the production of neurotransmitters such as 5-HT, dopamine, and norepinephrine. These neurotransmitters are transmitted to the brain by means of blood circulation or neural pathways and interact with vagal signalling to regulate the central nervous system, directly affecting mood and memory-related neurons. Cao *et al*. [10] found that there were significant differences in the composition of gut microbiota between patients with depression and healthy people at the phylum and genus levels. The enrichment of bacteria in the phylum *Actinobacteria* and *Proteobacteria*, as well as the families *Rikenellaceae* and *Bifidobacteriaceae*, is higher in depressed patients. In contrast, the abundance of *Firmicutes*, *Prevotellaceae* and *Ruminococcaceae*, is lower. Liu *et al*. [26] also found that the overall gut microbial composition of patients with depression is distinct from that of healthy controls, further confirming that intestinal microbial dysbiosis is an important feature of depression. Lai and Xiong [11] reported in a Mendelian randomization investigation that increased abundance of bacteria in the phylum *Actinobacteria* (especially *Bifidobacteriaceae*), *Firmicutes Clostridium*, and *Proteobacteria Parasutterella* had a protective effect against depression, whereas increased bacterial abundance of δ-*Proteobacteria*, *Desulfovibrioles* and *Oxalicobacterium* significantly augmented the risk of depressive symptoms. Wu *et al*. [27] found that *Bacteroides* and its metabolite p-hydroxyphenylacetic acid in feces could alleviate the depressive-like behaviour of a lipopolysaccharide-induced mouse model of depression. Guida *et al*. [28] showed that antibiotic-induced gut microbiota dysbiosis in mice leads to activation of systemic inflammation and depression-like behaviour, which can be reversed after mice are given the probiotic *Lactobacillus casei*. Moreover, FMT using samples from patients with major depressive disorder can trigger depression-like behaviors when performed in rodent subjects [29]. A 16S rRNA sequencing study by Zhou *et al*. [30] revealed that the gut microbiota profile of subjects with depressive disorders revealed a decrease in the abundance of beneficial bacteria, including *Bacteroides faecalis, Lactobacillus, Clostridium butyricum* and *Helicobacter* spp. that produce short-chain fatty acids (SCFAs), and an increase in the abundance of pro-inflammatory *Enterobacteriaceae*. In addition, lower abundance of *Phascolarctobacterium, Helicobacter* spp., *Bacteroides faecalis* and *Tyzzerella* was associated with greater severity of depressive symptoms. Numerous studies have indicated that the gut microbiota dysbiosis is closely associated with depression. However, the specific microbial taxa that contribute to disease susceptibility and progression still need further exploration. Clarifying depression-associated microbial signatures will have a profound impact on the prevention and treatment of depression.

### Potential Mechanisms by Which Gut Microbiota Affects Depression

#### Brain–Gut Peptide Pathway

Brain–gut peptides (BGP), as important regulators of the BGA system, are functional polypeptides produced by the brain and gastrointestinal tract, playing a core role in neuroendocrine, intestinal system and mood regulation [31]. Neuropeptide Y, gastrin-releasing peptide, auxin-releasing peptide, gastric inhibitory peptide and other BGPs have been confirmed to be intimately related to depressive symptom severity [32, 33]. The gut microbiota has the following mechanisms of influence on the BGP system: First, it directly stimulates intestinal endocrine cells by producing SCFAs and various other metabolites derived from the fermentation of dietary fibre. Promotes the synthesis and release of 5-HT precursor tryptophan and other BGPs [34]; secondly, the metabolites of the microbiota can enter the circulatory system and vagal neural pathways to exert effects on HPA axis function, and regulate the levels of cortisol and other stress-related hormones. Overactivation of the HPA axis is considered to be one of the core pathological links of depression [35]. Schaub *et al*. [12] showed that short-term supplementation with high-concentration multi-strain probiotics is capable of significantly mitigating depressive symptoms in patients, suggesting that the enhancement in the abundance of *Lactobacillus* in the gut is directly related to ameliorate depressive symptoms. This study further found through neuroimaging that probiotic intervention can reduce the neural reactivity of the brain to neutral emotional stimuli, and this process is precisely the intervention of the central nervous system by the gut microbiota through regulating the synthesis and secretion of BGPs.

#### Immune Inflammatory Pathway

The regulation of the host immune system by gut microbiota and the mediation of systemic inflammation are believed to contribute to the pathogenesis of depression. Gut microbiota dysbiosis may increase the abundance of pathogenic or opportunistic pathogens. Components of these pathogens, such as lipopolysaccharides, can disrupt the close connections between intestinal epithelial cells, thereby increasing intestinal permeability. This makes it easier for pro-inflammatory substances such as bacterial antigens to stimulate systemic immune responses through the circulatory system, thereby causing a persistent mild inflammatory state [36]. Studies have shown that patients with depression present markedly elevated levels of inflammatory factors including interleukin-6 and tumour necrosis factor-α compared with healthy individuals, with depression symptom severity showing a positive correlation with the levels of these mediators [37]. Peripheral inflammatory factors can enter the central nervous system (brain) by means of active transport and conduct afferent signals along the vagus nerve, or through weak areas of the blood–brain barrier, thereby affecting the central nervous system through systemic inflammatory responses. In addition, they can also activate microglia in the brain, prompting the central nervous system to produce its own inflammatory response. Sustained high levels of pro-inflammatory cytokines can directly interfere with neurotransmitter metabolism, such as activating indoleamine 2,3-dioxygenase, which shifts the tryptophan metabolic pathway from the synthesis of 5-HT to the synthesis of kynurenine, which can further generate neurotoxic quinolinic acid, damaging neurons and causing depressive symptoms [38]. Inflammation may also inhibit the expression of brain-derived neurotrophic factor (BDNF), impair the neuroplasticity of brain regions such as the hippocampus, and may exacerbate the hyperfunction of the HPA axis, constituting the inflammatory pathological basis of depression [39] .

#### Other Pathways

In addition to immune and inflammatory pathways, gut microbiota may also influence the pathophysiology of depression through metabolic and endocrine pathways. As an important “metabolic factory” of the human body, gut microbiota is responsible for the metabolism of many nutrients that the human body cannot synthesize on its own. Gut microbiota ferments dietary fibre to produce SCFAs (such as butyric acid and propionic acid). These substances can not only reduce systemic inflammation, but can also penetrate the blood–brain barrier, regulate the function of microglia, and upregulate the expression of BDNF in the hippocampal region, thereby affecting neuronal function and relieving depressive symptoms [40].

The HPA axis is the core endocrine pathway for mediating stress responses and mood regulation. Its dysfunction is the core pathology and important cause of depression. Intestinal microbiota and its derived metabolites, including SCFAs, can regulate HPA axis activity and affect the secretion of key stress hormones such as cortisol. A study has shown that HPA axis overactivation is common in patients with depression [41]. Specifically, the increased activity of corticotropin-releasing hormone and arginine vasopressin is significantly enhanced, and the negative feedback regulation function mediated by glucocorticoid receptor is impaired, which ultimately leads to the sustained high level of systemic stress hormones including cortisol. This persistent HPA axis activation has been associated with dysfunction of the hippocampus, impaired neuroplasticity, and the development of depressive symptoms [42].

## Treatment of Depression Based on the Gut Microbiota-Brain Axis Theory

### Probiotics or Prebiotics

Probiotics are a type of active microbial community that is beneficial to the physiological health of the host. Common probiotic genera include *Lactobacillus, Bifidobacterium*, as well as selected *Streptococcus* and *Enterococcus* species. Probiotic supplementation can regulate the species composition and abundance balance of the intestinal microbiota, enhance intestinal barrier function, regulate immune response, promote the digestion and absorption of nutrients, and inhibit the excessive proliferation of intestinal pathogens and maintain the homeostasis of the digestive tract. Nikolova *et al*. [13] conducted a randomized, double-blind, placebo-controlled trial enrolling 49 patients with depression. The patients received 8 × 10^9^ colony-forming unit (CFU) of probiotics daily for 56 consecutive days. The Hamilton Depression Rating Scale score of the probiotic group decreased significantly from baseline. The mean score was 8.83 points at week 8, compared with the placebo group. Another randomized controlled study on major depressive disorder showed [43] that high-dose probiotic adjuvant therapy could significantly improve the cognitive function of patients, especially the immediate recall ability in verbal episodic memory. Neuroimaging studies have shown that probiotic intervention can improve hippocampal function, which is closely related to memory and mood regulation. Mörkl *et al*. [44] conducted a randomized controlled trial recruiting 43 patients with depression. After taking probiotics, the patients’ morning vagal nerve function was significantly enhanced, and the abundance of beneficial bacteria such as *Akkermansia muciniphila* in the intestine increased, and the patients’ sleep quality was also significantly improved. This study suggests that probiotics may ameliorate patients’ depressive symptoms and improve physiological function by regulating the gut microbiota and stimulating the vagus nerve.

As indigestible food components, prebiotics are capable of selectively boosting the growth and metabolic functions of probiotics. For instance, oligosaccharides, fructans, and galactose not only enhance probiotic activity but also exert a positive regulatory effect on the structure of the host’s microbial community. An animal study indicated that in a high-fat diet-induced mouse model of obesity and depression-like phenotypes, supplementation with oligofructose and oligogalactose could effectively improve the depression- and anxiety-like behaviour of mice [45]. A randomized controlled study by Khademi *et al*. [46] on 51 obese women with mild depression involved daily calorie control combined with prebiotic supplementation for 8 weeks. The results showed that, compared with the placebo group, the prebiotic group exhibited slight improvements in serum tryptophan levels and depressive symptom scores; however, these between-group differences were not statistically significant. These findings suggest that probiotics and prebiotics may exert their antidepressant effects through multiple pathways, including regulating neurotransmitter precursors such as tryptophan, alleviating neuroinflammation, and modulating neural activity. Essentially, they establish a bridge between gut microbiota and central nervous system regulation via the BGA, paving new ground for non-pharmacological interventions in depression.

### FMT

FMT represents is an emerging microbiota-based therapeutic approach. The core procedure of this method is to extract and isolate beneficial gut microbial flora from the feces of healthy donor cohorts and then transplant them into the gastrointestinal tract of recipient individuals. This intervention aims to reconstruct the gut microbiota ecology of patients and provide a highly promising microecological treatment strategy targeting neuropsychiatric disorders including depression and anxiety through the core regulatory pathway of the BGA. Jiang *et al*. [14] reported that FMT was associated with improvements in depressive symptoms of patients, suggesting its potential therapeutic role in alleviating depression. An animal study showed that FMT can markedly elevate the sucrose preference rate of model animals and reduce immobility time in the forced swimming test, suggesting its direct antidepressant effect. In addition, the study also observed that FMT altered the gut microbiota in murine models, increased *Firmicutes* abundance, and improved the intestinal barrier function [47]. Hu *et al*. [48] transplanted the gut microbiota of healthy rats into depressed rats in an animal experiment and demonstrated the antidepressant effect of healthy microbiota. The recipient rats not only showed significant relief from depressive-like behaviours but also exhibited increased levels of neurotransmitters and decreased levels of pro-inflammatory cytokines in the hippocampus. FMT may modulate the multi-level pathways of the BGA by reshaping the gut microbiota, including restoration of the intestinal mucosal barrier, modulation of neuroactive metabolites, and regulation of systemic and neuroinflammatory levels. This influences the neuroplasticity and function of brain regions associated with mood regulation. Consequently, FMT represents an intervention strategy based on brain–gut bidirectional regulation, highlighting the scientific value of the BGA as a key therapeutic target for depressive. However, research on donor fecal microbiota selection and recipient tolerance is limited, and the effectiveness of FMT in treating depression requires further investigation.

### Drug Therapy

Many antidepressants and their active ingredients can regulate the gut microbiota composition and enhance intestinal barrier integrity, thereby alleviating depressive symptoms through the BGA. An animal study suggested that the non-absorbable antibiotic rifaximin can improve depressive-like behaviours in chronically stressed rats through modulation of the BGA [49]. It was also found that rifaximin can modulate the composition of gut microbiota, with a marked increase in the abundance of butyrate-producing bacteria such as *Ruminococcaceae* and *Lachnospiraceae*. Another study confirmed that berberine can inhibit systemic and neuroinflammatory responses and reduce corticosterone by reinstating gut microbiota diversity and elevating levels of SCFAs (particularly butyrate), and ultimately exerting a therapeutic antidepressant effect [50]. A meta-analysis confirmed that flavones from traditional Chinese medicine produced a significant ameliorative effect on diverse depressive-like phenotypes in mice and systematically regulated pathophysiological processes associated with depression, reduced oxidative stress, inhibited neuroinflammation, and downregulated corticosterone levels [51]. Chen *et al*. [15] reported that the traditional Chinese medicine Xiaoyao San may exert a therapeutic effect on post-stroke depression via multi-targeted regulation of the BGA. Oral administration of Xiaoyao San may inhibit the proliferative activity of pathogenic bacterial strains, alter the gut microbiota composition, and regulate the intestinal metabolite spectrum. In addition, active ingredients in Xiaoyao San have been reported to inhibit NOD like receptor pyridine domain containing 3 (NLRP3) inflammasomes in the colon and hippocampus and reduce systemic inflammatory responses. Zhu *et al*. [52] reported that Danzhi Xiaoyao San combined with SSRIs was associated with greater improvement in depressive symptoms than SSRI treatment alone. SSRI Danzhi Xiaoyao San therapy can significantly change the composition of the gut microbiota of patients and affect the levels of 39 serum metabolites, including lysophosphatidic acid. The studies discussed above indicate that a variety of pharmacological agents exert effects on the BGA through the gut microbiota. By reshaping the microbial ecosystem, these drugs modulate microbial metabolites, ultimately influencing central emotional and cognitive functions through improvements in the intestinal barrier, regulation of the HPA axis, and modulation of neurotransmitters. This reveals the central role of the BGA in the antidepressant mechanisms of drugs.

### Diet

Recently, the intersection of nutritional science and psychiatry has attracted increasing attention. Related studies have clarified the potential of diet to regulate mental state and provided new ideas for the comprehensive management of mental health. Dietary patterns can affect the BGA function, thereby changing the host’s homeostasis. A randomized clinical study in patients with type 2 diabetes showed that a high-fibre diet can reduce the depression score and alter gut microbiota composition. Dietary intervention significantly increased the abundance of commensal beneficial bacteria such as lactobacillus and bifidobacteria in patients and simultaneously improved serum metabolism and reduced systemic inflammation [53]. A prospective randomized controlled trial for patients with moderate to severe depression included 67 subjects who received 12 weeks of Mediterranean diet guidance [16]. The results showed that the depression symptoms (Montgomery-Asberg Depression Rating Scale (MADRS) score) declined, which suggests that dietary improvement achieved through nutritional guidance may alleviate depression symptoms by regulating gut microbiota and other pathways. In another 12-week randomized controlled trial involving 72 depressed patients who received the Mediterranean diet intervention showed significant and substantial improvements in dietary adherence, depressive symptoms (Beck Depression Inventory-II (BDI-II) score), and quality of life compared with controls [54]. The aforementioned studies suggest that nutritional interventions directly shape gut microbiota composition by adjusting dietary patterns (e.g., high-fiber, Mediterranean diets), increasing beneficial bacterial abundance, and improving metabolic and inflammatory states. These changes integrate through the BGA to regulate neuroendocrine and immune pathways, ultimately achieving relief from depressive symptoms.

### Exercise

Increasing evidence suggests that physical exercise can not only boost physical fitness and lessen the burden of illness, but also positively affect mental health. In fact, physical exercise might confer potential benefits for a variety of neurological and psychiatric disorders, including major depressive disorder. A meta-analysis by Pearce *et al*. [17] found that physical exercise had significant benefits in reducing the risk of depression, even in countries with poor public health. Another systematic review and meta-analysis also concluded that physical exercise had a positive effect on depressive symptoms in people of all ages [55]. A randomized controlled trial of 20 healthy adults showed that regular resistance training significantly improved mood and reduced serum lipopolysaccharide-binding protein levels [56]. These studies suggest that resistance exercise can reduce systemic inflammation levels by maintaining intestinal barrier integrity and regulating gut microbiota and metabolic status. These physiological changes undergo signalling integration via the BGA, thereby modulating neuroendocrine function and ultimately exerting positive effects on mood and cognitive function. This provides mechanistic evidence for exercise improving depression through the BGA.

## Conclusions

Accumulating evidence suggests that the BGA may serve as a central link between the gut microbiota and the onset of depression. This paper incorporates several key studies that provide robust evidence—covering aspects such as the characteristics of microbiota dysbiosis and the efficacy of interventions—confirming the critical role of the gut microbiota in the onset and progression of depression. It reinforces the theory of “bidirectional brain–gut regulation” and provides preliminary validation of the feasibility of targeting the gut microbiota for intervention.

However, current research still has certain limitations. The specific molecular mechanisms by which the BGA regulates depression remain unclear. In addition, this review did not explore the relationship between different depression subtypes and the BGA. Intervention studies are limited by small sample sizes and short follow-up periods, whilst the safety and applicability of novel intervention methods require further validation. Furthermore, the lack of uniform research standards makes it difficult to compare results across studies.

Future research should focus on these gaps, further elucidating the regulatory targets of the BGA, conducting large-scale, long-term randomised controlled trials to optimise intervention protocols, establishing standardised research protocols, and identifying specific microbial biomarkers to facilitate the translation of basic research into clinical practice. Overall, the plasticity of the gut microbiota offers a new avenue for depression intervention; future efforts must address these research gaps to bridge the gap between exploratory correlations and clinical application.

## Availability of Data and Materials

This review involves no original raw data, and all relevant evidence is extracted from published studies.
